# A new name for an old problem—*Colletotrichum cigarro* is the cause of St John’s wilt of *Hypericum perforatum*

**DOI:** 10.3389/ffunb.2024.1534080

**Published:** 2025-01-23

**Authors:** Lana-Sophie Kreth, Ulrike Damm, Monika Götz

**Affiliations:** ^1^ Institute for Plant Protection in Horticulture and Urban Green, Julius Kühn-Institute (JKI) – Federal Research Centre for Cultivated Plants, Braunschweig, Germany; ^2^ Department of Botany, Senckenberg Museum of Natural History Görlitz, Görlitz, Germany

**Keywords:** anthracnose, *Colletotrichum cigarro*, *Colletotrichum gloeosporioides*, *Colletotrichum kahawae*, *Hypericum perforatum*, *Glomerella cingulata* var. *migrans*, St John’s wilt pathogen, St John’s wort

## Abstract

A major problem for St John’s wort (*Hypericum perforatum*) is St John’s wilt, which can lead to reduced crop yields and even complete crop losses. In the past, the pathogen was referred to as *Colletotrichum gloeosporioides* or occasionally as *Colletotrichum* cf. *gloeosporioides* based on morphology. Although a strain from this host had been re-identified as *C. cigarro* in taxonomic studies, there is uncertainty about the identity of the St John’s wilt pathogen, which is generally still addressed as *C. gloeosporioides* in applied science. In a multi-locus [internal transcribed spacer (ITS), glyceraldehyde-3-phosphate dehydrogenase (GAPDH), actin (ACT), and glutamine synthetase (GS)] analysis of the *C. gloeosporioides* species complex, all isolates obtained from newly collected symptomatic *H. perforatum* stems and seeds from Germany and Switzerland were identified as *C. cigarro.* Although they belonged to the same haplotype, the morphology of the isolates was very variable. Pathogenicity tests demonstrated that only *C. cigarro* strains from *H. perforatum* cause symptoms on *H. perforatum*, whereas other *Colletotrichum* species tested only caused latent infection of *H. perforatum*.

## 1 Introduction

St John’s wort (*Hypericum perforatum* L.) is an important medicinal plant with a wide range of applications ranging from nervousness, restlessness, anxiety, to depression, as well as external treatment of injuries or burns (Bomme, 1997). Recently, new application areas are being investigated including treatment of cancer and Alzheimer’s disease (Choudhary et al., 2022; Yuan et al., 2023; Suryawanshi et al., 2024). The demand for St John’s wort drugs is met by imports from various countries, partly by wild collections (Fachagentur Nachwachsende Rohstoffe e.V, 2014). Today, St John’s wort cultivation is estimated at approximately 150 ha in Germany. *H. perforatum* is attacked by various fungal pathogens causing powdery mildew (*Erysiphe hyperici*), leaf blight (*Diploceras hypericinum*), wilt (*Colletotrichum* cf. *gloeosporioides* and *Verticillium* spp.), and leaf spot (*Alternaria alternata* and *Stemphylium botryosum*) diseases (Putnam, 2000; Fritzsche and Hoppe, 2007).

In 1937, a fungal pathogen was isolated from severe stem infection of *H. perforatum* collected in the Botanical Garden Berlin-Dahlem, Germany, and initially identified as *Gloeosporium orbiculare* Berk. et. Mart. Based on the subsequently formed sexual morph, the fungus was described as *Glomerella cingulata* var. *migrans* Wollenw (Wollenweber and Hochapfel, 1949). In the 1990s, the cultivation of *H. perforatum* in Germany suffered from considerable yield losses and even total losses due to St John’s wilt (Plescher, 1997; Gärber, 1999), and first occurrences of this disease were also reported from other European countries (Schwarczinger and Vajna, 1998; Debrunner et al., 2000; Filoda, 2004). The pathogen was morphologically identified by Gärber (1999) and referred to as *C.* cf. *gloeosporioides* Penz. (anamorph, teleomorph: *G. cingulata* var. *migrans*). The St John’s wilt pathogen initially causes anthracnose at the stem base, resulting in drooping shoot tips and later in wilting of the plant (Hildebrand and Jensen, 1991; Schwarczinger and Vajna, 1998; Gärber, 1999; Debrunner et al., 2000; Filoda, 2004). Shepherd (1995) showed that the pathogen is host-specific. The pathogen spreads from plant to plant by conidia in a short period of time (Gärber and Schenk, 2002a). Infection of buds, flowers, and seeds has been demonstrated, but the pathogen is primarily regarded as seed-borne (Gärber and Schenk, 2002b). Since *H. perforatum* is cultivated in the field for 2–3 years, the pathogen can overwinter in the plant population, which happens by means of ascospores (Gärber and Schenk, 2002a, 2002b). St John’s wilt still poses a major problem for the commercial cultivation of *H. perforatum* (Wahl et al., 2018).

In 2012, Weir et al. (2012) performed a comprehensive phylogenetic study of the *Colletotrichum gloeosporioides* species complex on the basis of up to eight nuclear gene regions and accepted 22 species, one of them with two subspecies. In their phylogeny, *C. kahawae* that had been described by Waller et al. (1993) for strains causing coffee berry disease (CBD) clustered with strains from several hosts other than coffee. Therefore, *C. kahawae* J.M. Waller & Bridge was reduced to subspecies level, *C. kahawae* subsp. *kahawae* J.M. Waller & Bridge, while *C. kahawae* subsp. *cigarro* B.S. Weir & P.R. Johnst. (as “*ciggaro*”) was established for strains from other hosts. These strains also included the ex-type strain of *G. cingulata* var. *migrans* (CBS 237.49) from *H. perforatum* in Germany; this variety was therefore synonymized with the latter subspecies. Of the loci used in the study of Weir et al. (2012), only the presence/absence of a 22-bp-long deletion in the GS gene could distinguish between the two subspecies. Moreover, the CBD strains are not able to utilize citrate or tartrate, whereas strains of *C. kahawae* subsp. *cigarro* are able to use one or both of these carbon sources (Waller et al., 1993). Cabral et al. (2020) recently resurrected *C. kahawae* and raised *C. kahawae* subsp. *cigarro* to species rank, as *C. cigarro* (B.S. Weir & P.R. Johnst.) A. Cabral & P. Talhinhas, which also includes the ex-type strain of *G. cingulata* var. *migrans*. Until now, it has not been investigated if the *Colletotrichum* strains causing St John’s wilt in commercial fields in Europe since the 1990s represent the same species as strain CBS 237.49 isolated almost 90 years ago from stem infection of *H. perforatum* in the Botanical Garden Berlin-Dahlem that originally had been described as *G. cingulata* var. *migrans.* As no studies have previously been conducted on the St John’s wilt pathogen based on modern systematics of the genus *Colletotrichum*, the identity of the pathogen needs to be clarified. Therefore, the aim of this study is to determine (1) if this disease is caused by one or more *Colletotrichum* species; (2) which species occur(s), if the (main) pathogen is *C. cigarro*; and (3) if there is a molecular or morphological variability within strains of the pathogen(s). This will be investigated based on multi-locus sequence and morphological data of isolates from St. John’s wilt from commercial and experimental fields, as well as roadside flora in Germany and Switzerland and several culture collections. A pathogenicity test will be carried out to confirm the pathogenicity of the species to *H. perforatum*.

## 2 Materials and methods

### 2.1 Sample collection and fungal isolation

Symptomatic plants of *H. perforatum* were collected at different locations in Germany and Switzerland from commercial fields (two sites in Groß Schierstedt, Germany; two sites in Uttwil, Switzerland), experimental fields (Braunschweig and Quedlinburg, Germany), and roadside flora (two sites in Braunschweig) in 2021 and 2022. In addition, nine seed lots provided by commercial distributors were included in this study.

After macroscopic and microscopic (Axio Imager.A1 equipped with an AxioCam MRc5 camera, Zeiss, Germany) inspection, plant materials were washed with tap water to remove the soil particles. Seeds and approximately 3-cm-long stem segments were surface sterilized with sodium hypochlorite solution (1% active chlorine) containing 0.1% Tween^®^ 20 (Carl Roth, Germany) for 2 min. After two washing steps in sterile water, the plant materials were dried on sterile filter paper and pressed on potato dextrose agar (PDA, Becton Dickinson, New Jersey, USA) to check surface sterilization. Afterwards, stem segments were cut into 0.5-cm pieces and halved lengthwise. Stem pieces and seeds were transferred to PDA containing antibiotics [100 mg/L penicillin G sodium salt (PEN–NA, Merck, Germany), 10 mg/L chlortetracycline hydrochloride (C4881, Merck, Germany), and 50 mg/L streptomycin sulfate salt (S6501, Merck, Germany)]. Cultures were incubated under natural daylight at 20°C and inspected daily for mycelial growth. After 3–5 days, mycelia producing Colletotrichum-like spores were observed. Outgrowing hyphae of these colonies were transferred to new PDA plates. Single conidial isolates were prepared from the sporulating cultures by streaking a few spores on new PDA plates with an inoculation loop. Plates were incubated at 20°C in the dark and examined for germinating conidia under an inverted microscope (Primovert, Zeiss, Germany). Single germinating conidia were cut out, transferred to new PDA plates, and incubated as described before (Götz et al., 2024).

For storage in the institute’s culture collection, the strains were grown on PDA supplemented with 5% (v/v) glycerol (Carl Roth, Germany) for 2 weeks at 20°C in the dark. Mycelial plugs were cut from the border of the growing colonies, transferred to Nalgene^®^ cryotubes (Thermo Scientific, Germany) containing potato dextrose broth (PDB, Becton Dickinson, New Jersey, USA) amended with 15% (v/v) glycerol, and frozen at -70°C in a Nalgene^®^ Mr. Frosty^®^ freezing container (Thermo Scientific, Germany). Selected strains (JKI-GF-Z1952, JKI-GP-23-019, and JKI-GP-23-020) were deposited at the Leibniz Institute – German Collection of Microorganisms and Cell Cultures GmbH, Germany (DSMZ).

To include further *Colletotrichum* strains from *H. perforatum* in this study, several culture collections worldwide [DSMZ, Mycotheque de l’Universite catholique de Louvain, Belgium (BCCM/MUCL), the Westerdijk Fungal Biodiversity Institute, The Netherlands (CBS), the CABI Genetic Resource Collection, United Kingdom (CABI), the American Type Culture Collection, Virginia, USA (ATCC), the International Collection of Microorganisms from Plants, New Zealand (ICMP), and the Institute of Epidemiology and Pathogen Diagnostics of the JKI, Germany (JKI-EP)] were searched. Three additional strains were found, JKI-EP-70790, JKI-EP-71555, and CBS 237.49. Additionally, three strains of *C. gloeosporioides* were included.

### 2.2 DNA extraction, PCR amplification, and sequencing

Fungal DNA was extracted from mycelium grown on PDA at 20°C in the dark using the DNeasy Plant Mini Kit (Qiagen, Germany) with the following modifications: Approximately 80 mg of mycelium was homogenized with two steel beads (5 mm diameter) and 400 µL of AP1 buffer (Qiagen, Germany) in 2-mL tubes using a bead mill (MM400, Retsch, Germany) at a frequency of 27 Hz for 5 min. After addition of 4 µL of RNase, the samples were incubated at 60°C ± 2°C and 1,000 rpm for 60 min (Thermomixer comfort, Eppendorf, Germany). The subsequent steps were carried out following the manufacturer’s protocol.

Four DNA loci were amplified: The 5.8S nuclear ribosomal gene with the two flanking internal transcribed spacers (ITS), a 200-bp intron of the glyceraldehyde-3-phosphate dehydrogenase (GAPDH), and partial sequences of the actin (ACT) and glutamine synthetase (GS) genes. The primer sets used are listed in **Table 1**. The PCR mix contained 10 µL of 5 × HOT FIREPol^®^ Blend Master Mix with 7.5 mM MgCl_2_ (Solis Biodyne, Estonia), 5 µL (ITS, GAPDH) or 2 µL (ACT, GS) of each primer (10 µM), and 10 µL (ITS, GAPDH) or 4 µL (ACT, GS) of genomic DNA and was filled up to a total volume of 50 µL with sterile water. The PCR conditions were 13 min at 95°C, followed by 35 cycles of 30 s at 95°C, 30 s at annealing temperature (ITS: 53°C, GAPDH: 60°C, ACT: 58°C, GS: 54°C), 45 s (GAPDH, ACT, and GS) or 60 s (ITS) at 72°C, and then 7 min (GAPDH and ACT) or 10 min (ITS and GS) at 72°C. The PCR products were purified using the DNA Clean & Concentrator Kit (Zymo Research Europe GmbH, Germany) and sequenced in forward and reverse direction using the same primers as used for amplification (Microsynth Seqlab GmbH, Germany). Consensus sequences were generated and manually edited when necessary using CLC Main Workbench 23.0.1 (Qiagen, Germany) following the EPPO recommendations for sequence analysis (OEPP/EPPO, 2021). All consensus sequences generated in this study were deposited in NCBI GenBank (https://www.ncbi.nlm.nih.gov/genbank/). The accession numbers are listed in **Supplementary Table 1**.

**Table 1:** Primer combinations used in this study for PCR amplification and sequencing.

Locus	Primer	Sequence (5′−3′)	Reference
ITS	ITS1	TCCGTAGGTGAACCTGCGG	White et al., 1990
	ITS4	TCCTCCGCTTATTGATATGC	White et al., 1990
GAPDH	GDF	GCCGTCAACGACCCCTTCATTGA	Guerber et al., 2003
	GDR	GGGTGGAGTCGTACTTGAGCATGT	Guerber et al., 2003
ACT	ACT-512F	ATGTGCAAGGCCGGTTTCGC	Carbone and Kohn, 1999
	ACT-783R	TACGAGTCCTTCTGGCCCAT	Carbone and Kohn, 1999
GS	GSF1	ATGGCCGAGTACATCTGG	Guerber et al., 2003
	GSR1	GAACCGTCGAAGTTCCAG	Guerber et al., 2003

### 2.3 Phylogenetic analysis

BLASTn searches on NCBI GenBank were performed with the newly generated ITS consensus sequences for preliminary identification and selection of reference strains. In addition to the sequences of the reference strains, sequences of further *Colletotrichum* isolates and isolates from *Hypericum* spp. were included (Nirenberg et al., 2002; Staňková et al., 2014; Samaga and Rai, 2016; Ding et al., 2023; https://www.ncbi.nlm.nih.gov/genbank/; **Supplementary Table 1**). The phylogenetic analysis included 39 (ITS, GAPDH, and ACT) and 31 (GS, multi-locus) related species. The sequences of each locus were aligned separately using the online version of MAFFT v. 7.526 (https://mafft.cbrc.jp/alignment/server/; last visited 17 October 2024) adopting the iterative refinement method L-INS-i for the sequences of ITS, G-INS-i for those of GAPDH and GS, and E-INS-i for those of ACT (Katoh and Standley, 2013; Katoh et al., 2019). The other settings were default. Selection of the best-fit models and the phylogenetic analyses of the single-locus as well as the concatenated alignments were performed in MEGA X (Kumar et al., 2018). The default settings were used to find the best-fit model in the model selection tool, except for a partial deletion of gaps that was set to a cutoff of 95% and five threads. The phylogenetic trees were inferred by maximum likelihood (ML) with 1,000 bootstrap replications, the best-fit model, and a partial deletion of gaps with 95% coverage cutoff and other settings as default. *Colletotrichum fioriniae* (CBS 128517) was used as outgroup. The alignments and respective phylogenetic trees were deposited in Figshare (doi: 10.6084/m9.figshare.27315027).

### 2.4 Morphological characterization

To compare the growth of the *Colletotrichum* strains, mycelia from actively growing cultures on PDA were cut with a cork borer (0.7 cm diameter) and placed in the center of a new PDA plate (*n* = 3). The cultures were incubated at 20°C in the dark. After establishment of fungal growth on the new medium, the colony borders were marked on the four axes of a cross drawn on the bottom of the petri dish. After 7 days, the colony borders were marked again, the distances between each of the two marks were measured, and the mean value of the measurements was calculated for each strain.

The morphological characteristics of the *Colletotrichum* strains were examined using a standard light microscope (Axio Imager.A1 equipped with an AxioCam MRc5 camera). Measurements were made and images were taken with the calibrated ZEN Blue Edition software release 3.0 (Zeiss, Germany). The conidial length and width (*n* = 30) of 7-day-old cultures on PDA were analyzed following Frank (1990) with modifications: the five values indicate the minimum value, lower limit, arithmetic mean, upper limit, and maximum value, respectively; lower and upper limits indicate the range of 90% of all values.

### 2.5 Pathogenicity tests

Pathogenicity tests were conducted with 6-week-old seedlings and 3-week-old rooted head cuttings of *H. perforatum* (HO336, Jelitto Staudensamen GmbH, Germany) in a climate-controlled greenhouse. Seeds were sown in clay substrate (Klasmann-Deilmann, Germany) and grown at 18°C with a photoperiod of at least 14 h daylight. When daylight was insufficient, supplemental light was provided by sodium vapor lamps. Three weeks after sowing, seedlings were transplanted to multi-pot trays (77 pots per tray) filled with the same substrate and grown under the same conditions for another 3 weeks. Head cuttings from 4- to 5-month-old mother plants were cut and transferred directly to multi-pot trays filled with the same substrate and cultured at the same conditions as the seedlings. Three weeks after transfer, the head cuttings had rooted. For inoculum preparation, *Colletotrichum* strains from *H. perforatum* (JKI-GF-Z1952, JKI-GP-23-019, JKI-GP-23-020, and CBS 237.49) and other hosts (CBS 119204, DSM 62136, and DSM 62146) were incubated on PDA at 20°C in the dark for 2 weeks. Spore suspensions were prepared by applying 10 mL of sterile water to each Petri dish and sweeping off the spores with a sterile Drigalski spatula. The suspension was filtered through three layers of cheese cloth to remove mycelium and adjusted to a final concentration of 1 × 10^6^ spores mL^−1^ using a Neubauer improved counting chamber (Assistent, Karl Hecht GmbH & Co KG, Germany). Twenty head cuttings and seedlings, respectively, were inoculated with spore suspensions of each fungal strain by spraying them dripping wet. The same number of head cuttings and seedlings sprayed with sterile water served as non-inoculated control plants. All plants were incubated separately in small indoor greenhouses Maximus 3.0 (Meyer, Germany) at 20°C at a relative humidity of >80% and a photoperiod of 14 h in order to avoid cross-infection. To check germination, the spore suspensions of each strain were also sprayed onto PDA, incubated at 20°C in the dark, and checked after 1 to 2 days using an inverted microscope (Primovert, Zeiss, Germany). Inoculated plantlets were inspected regularly for the beginning of symptom development. After 7 and 14 days, the severity of symptoms was evaluated. Plant material from each treatment was taken randomly 7 days after inoculation and used for re-isolation of the previously inoculated fungus as described above. The experiment was repeated twice.

## 3 Results

### 3.1 Field symptoms and fungal isolates

**Figure 1:**
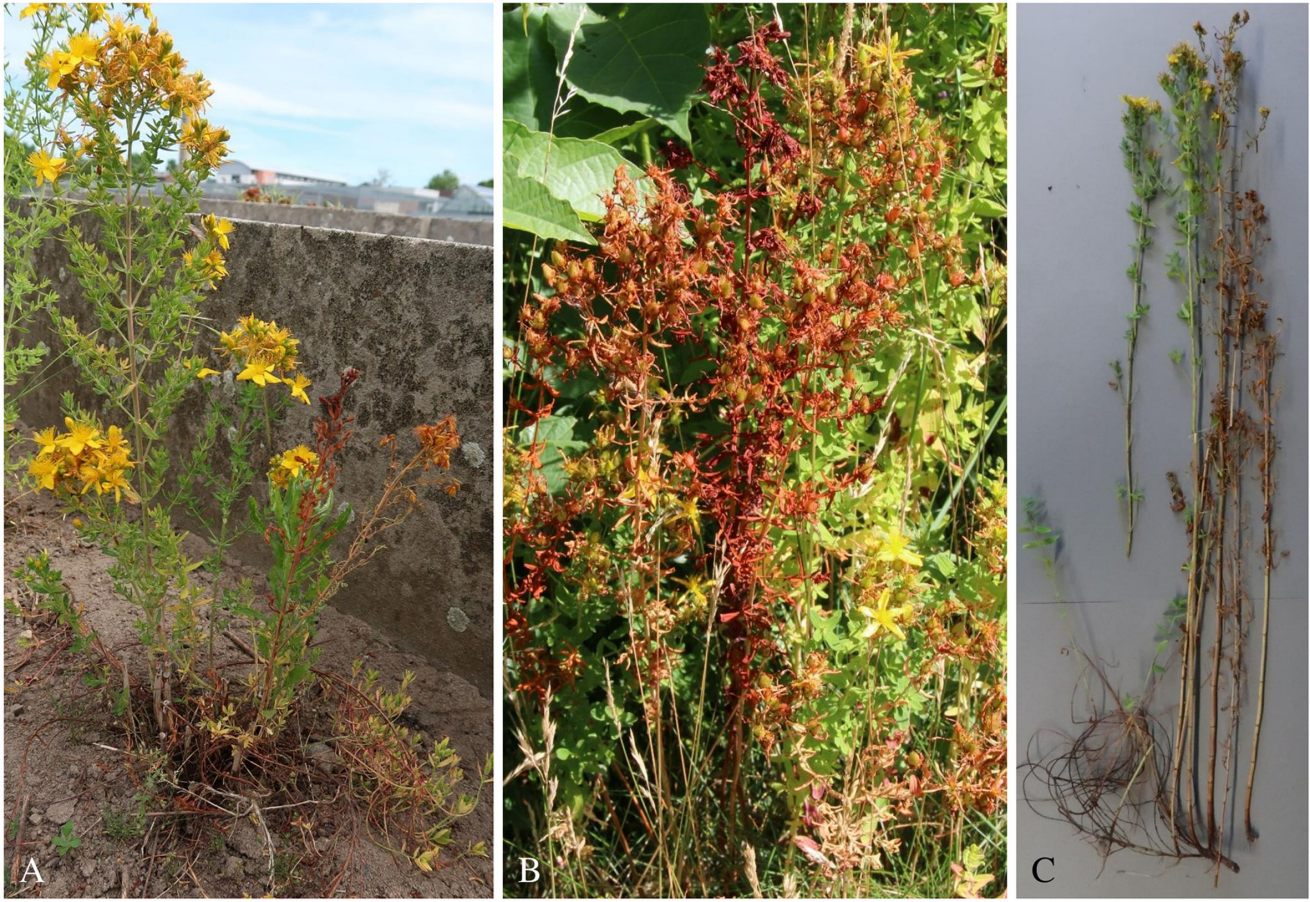
St John**’**s wilt symptoms on *Hypericum perforatum* **(A)** in an experimental field plot, **(B)** on the roadside, and **(C)** dug up from an experimental field plot.

On naturally infected plants, symptoms first appeared as small brown necrotic spots that developed at the stem base and spread over the entire stem base over time. In addition, drooping shoot tips, wilting symptoms, and even completely wilted plants were observed (**Figure 1**). Spores were not found on symptomatic tissue in the field. Infected seed did not differ morphologically from pathogen-free seed. *Colletotrichum* spp. was always isolated from symptomatic stem bases. However, it was only isolated from seeds of two seed lots. A total of 33 isolates of *Colletotrichum* spp. were recovered. From each location and seed lot, one representative isolate was selected and single conidial isolates were prepared for further molecular and morphological analysis (**Table 2**).

**Table 2:** *Colletotrichum* strains used in this study.

Species	Strain number	Host	Year of isolation	Location
*C. cigarro* ^a^	JKI-GF-Z1952, (DSM 116563)	*Hypericum perforatum*	1999	Germany, Kleinmachnow, roadside flora
*C. cigarro* ^a^	JKI-GP-23-030	*Hypericum perforatum*	2021	Switzerland, Uttwil, commercial field
*C. cigarro* ^a^	JKI-GP-23-019, (DSM 117132)	*Hypericum perforatum*	2021	Switzerland, Uttwil, commercial field
*C. cigarro* ^a^	JKI-GP-23-031	*Hypericum perforatum*	2021	Germany, Groß Schierstedt, commercial field
*C. cigarro* ^a^	JKI-GP-23-020 (DSM 116564)	*Hypericum perforatum*	2021	Germany, Groß Schierstedt, commercial field
*C. cigarro* ^a^	JKI-GP-24-010	*Hypericum perforatum*	2022	Germany, Braunschweig, experimental field
*C. cigarro* ^a^	JKI-GP-24-011	*Hypericum perforatum*	2022	Germany, Quedlinburg, experimental field
*C. cigarro* ^a^	JKI-GP-24-012	*Hypericum perforatum*	2022	Germany, Braunschweig, roadside flora
*C. cigarro* ^a^	JKI-GP-24-013	*Hypericum perforatum*	2022	Germany, Braunschweig, roadside flora
*C. cigarro* ^a^	JKI-GP-24-014	*Hypericum perforatum*	2022	Germany, seed lot
*C. cigarro* ^b^	JKI-EP-70790	*Hypericum perforatum*	1998	Germany, Bernburg
*C. cigarro* ^b^	JKI-EP-71555	*Hypericum perforatum*	2000	Germany, Quedlinburg
*C. cigarro* ^c^	CBS 237.49	*Hypericum perforatum*	1937	Germany, Berlin
*C. gloeosporioides* sensu stricto^d^	DSM 62136	*Citrus* sp. L.	Unknown	Italy
*C. gloeosporioides* s. str.^d^	DSM 62146	*Citrus sinensis* Pers.	Unknown	Greece
*C. gloeosporioides* s. str.^c^	CBS 119204	*Pueraria lobata* Willd.	2010	USA

a Isolated in this study or formerly by staff of the Institute for Plant Protection in Horticulture and Urban Green, JKI, Germany.

b Obtained from the strain collection of the Institute for Epidemiology and Pathogen Diagnostics, JKI, Germany.

c Obtained from Westerdijk Fungal Biodiversity Institute, The Netherlands.

d Obtained from the Leibniz Institute DSMZ − German Collection of Microorganisms and Cell Cultures GmbH, Germany.

### 3.2 Phylogenetic analysis

Initial BLASTn searches with newly generated ITS sequences of strains isolated from *H. perforatum* on NCBI GenBank resulted in matches with species of the *C. gloeosporioides* complex. Therefore, the newly generated DNA sequences of 12 *Colletotrichum* strains from *H. perforatum* and those of three *C. gloeosporioides* strains from other hosts were analyzed together with 32 reference strains that comprised all species of the *C. gloeosporioides* complex in Weir et al. (2012) as well as recently described species belonging to the Kahawae clade of this complex that had been selected based on BLASTn searches in NCBI GenBank (https://www.ncbi.nlm.nih.gov/genbank/ **Supplementary Table 1**). Additionally, 10 previously published *Colletotrichum* sequences of strains isolated from *Hypericum* species in Canada, China, Czech Republic, India, and Germany that were not available to us were retrieved from GenBank and included in the ITS, one of them also in the GAPDH single-locus analyses (Nirenberg et al., 2002; Ellsworth et al., 2013; Staňková et al., 2014; Samaga and Rai, 2016; Ding et al., 2023).

The alignments of the individual DNA regions were trimmed and then comprised 552 bp for ITS, 248 bp for GAPDH, 247 bp for ACT, and 975 bp for GS. The gene boundaries in the multi-locus alignment of the *C. gloeosporioides* species complex were as follows: ITS: 1–552, GAPDH: 555–802, ACT: 805–1,051, and GS: 1,054–2,028. The best models suggested by the model selection tool in MEGA X for the single-locus alignments were K2+G for ITS and ACT, K2 for GAPDH, T92+I for GS, and TN93+G for the multi-locus alignment (Kimura, 1980; Tamura, 1992; Tamura and Nei, 1993). A total of 428 positions were used in the ML single-locus analysis for ITS, 216 positions for GAPDH, 225 positions for ACT, 718 positions for GS, and 1,667 positions in the multi-locus analysis.

**Figure 2:**
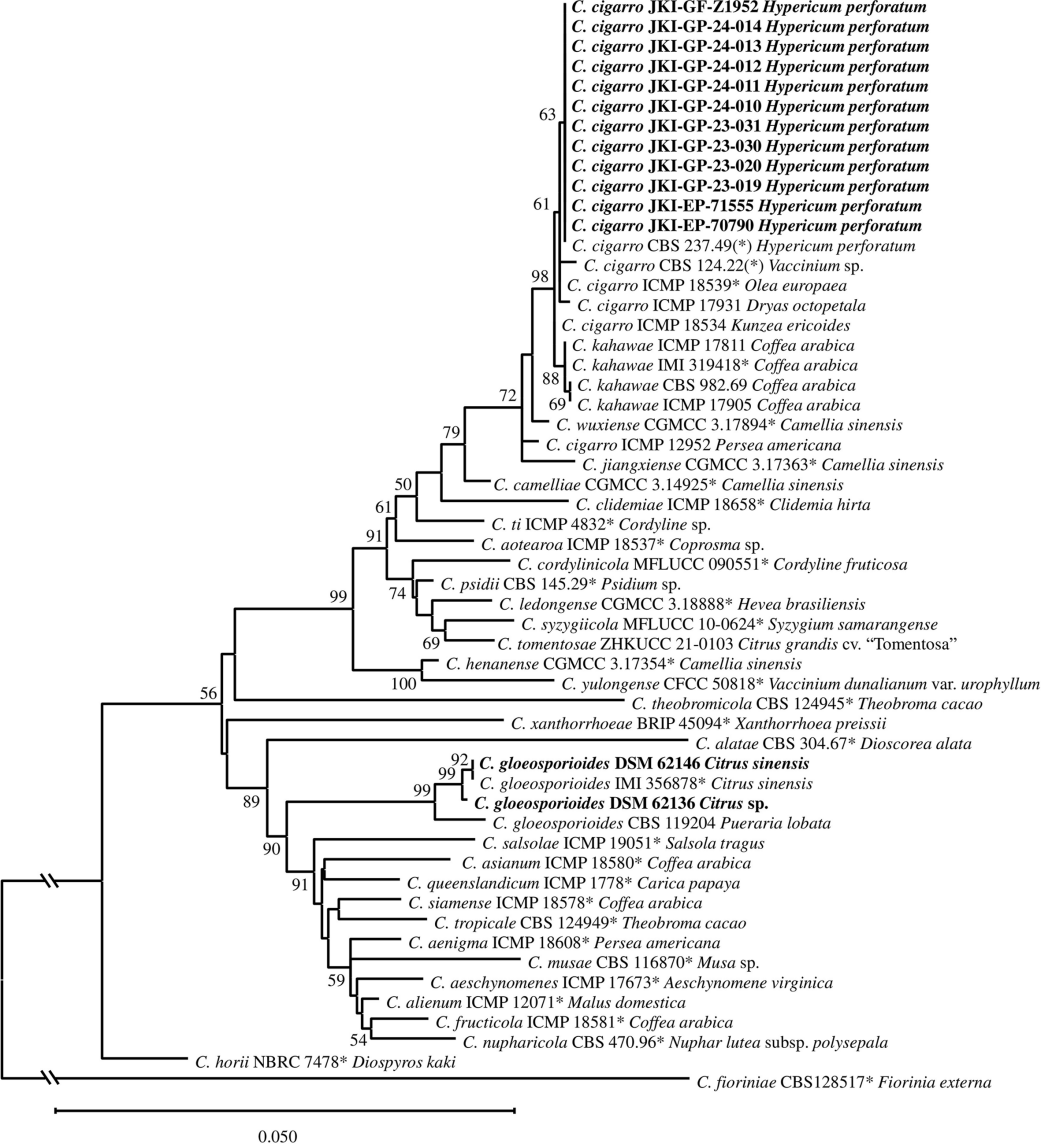
Maximum likelihood phylogenetic tree based on ITS, GAPDH, ACT, and GS sequences of *Colletotrichum* species in the *C. gloeosporioides* species complex. Numbers above branches show bootstrap values ≥50. Sequences obtained in this study are shown in bold. Ex-type strains are emphasized with an asterisk. Strain numbers are followed by host plant. Scale bar: number of substitutions per site.

In the multi-locus phylogeny (**Figure 2**), the strains from *H. perforatum* formed one uniform clade, but with a low bootstrap support (63%) due to the identical sequences of the *H. perforatum* strains and their small differences to *C. cigarro* strains from other hosts. This clade resides within a clade with the ex-type strain of *C. cigarro* (61%), which is sister to a clade containing all *C. kahawae* strains including its ex-type (88%). *Colletotrichum cigarro* ICMP 18534 was intermediate between the two sister clades within a larger clade with 98% bootstrap support, while *C. cigarro* ICMP 12952 and the ex-type strain of *C. jiangxiense* were outside this large clade, but grouping with it (72%). The systematic relationships between *C. jiangxiense*, *C. wuxiense*, and certain *C. cigarro* strains, especially ICMP 12952 and ICMP 18534, are not clear and need to be investigated in more detail elsewhere. Three strains from other hosts included in this study grouped with the ex-type of *C. gloeosporioides* with strong bootstrap support (99%).

It was not possible to assign the strains from *H. perforatum* to either *C. kahawae* or *C. cigarro* based on their ITS, GAPDH, or ACT sequences (**Supplementary Figures 1**-**3**). In the ITS and GAPDH phylogenies, the sequences of these strains clustered with those of several species of the *C. gloeosporioides* complex, including *C. cigarro* and *C. kahawae*. In the ACT phylogeny, the isolates from *H. perforatum* formed a clade with *C. helleniense* (CBS 142418) with low support (58%) within a large clade formed by all *C. cigarro*, *C. kahawae*, and other *Colletotrichum* isolates (63%). In the GS tree, all *C. kahawae* strains including its ex-type formed a subclade (88%), while all isolates from *H. perforatum* formed another subclade with the ex-type strain of *C. cigarro* and further *C. cigarro* isolates (64%); all of them forming a well-supported (99%) big clade with two further *C. cigarro* isolates and the ex-type strains of *C. camelliae* and *C. wuxiense* (**Supplementary Figure 4**).

### 3.3 Morphological characterization

**Figure 3:**
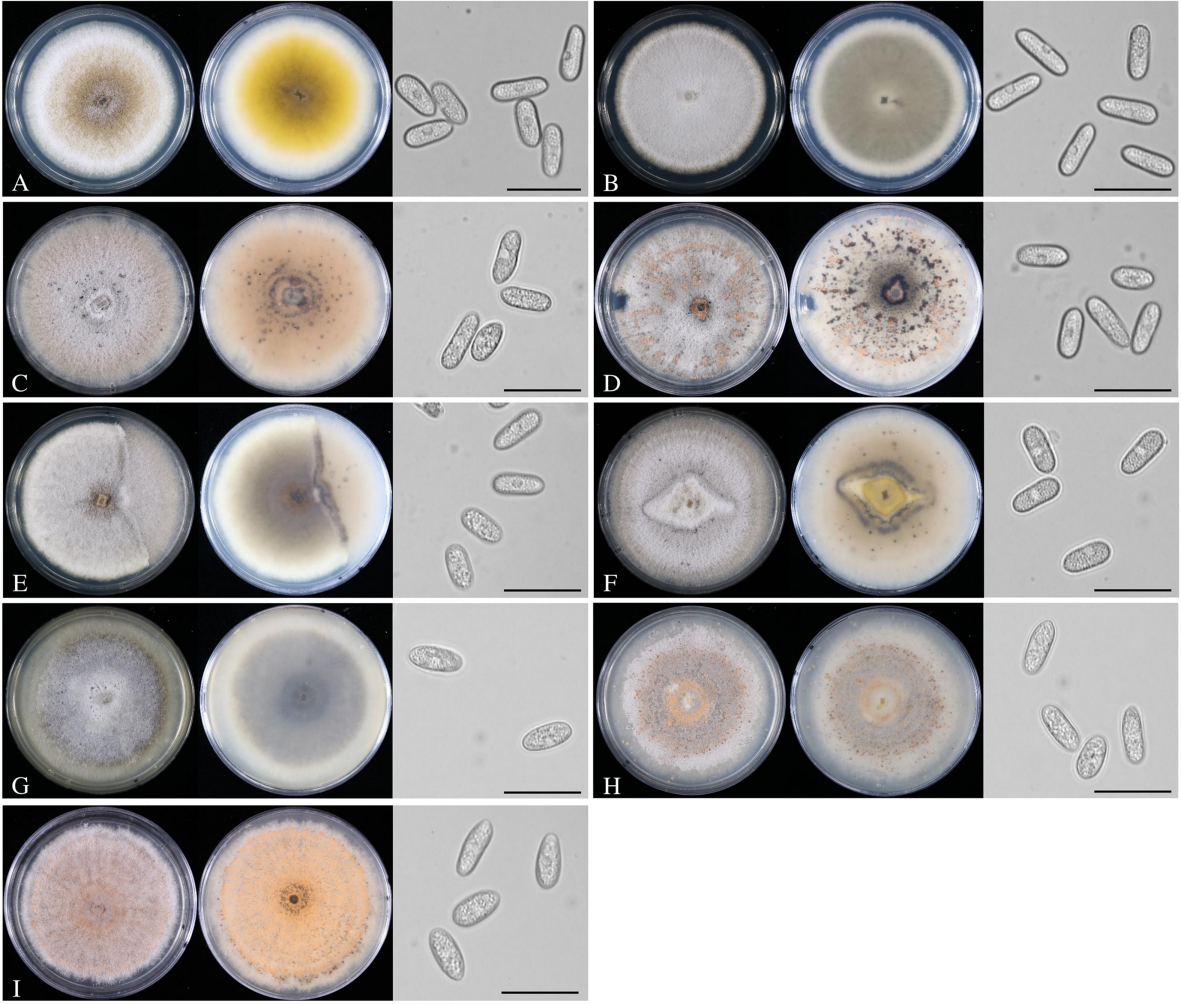
Colony morphology and conidia of *Colletotrichum cigarro* and *C*. *gloeosporioides* on PDA after 2 weeks in the dark at 20°C; top view (left), bottom view (middle), and spores (right). **(A)** *C*. *cigarro* JKI-GF-Z1952; **(B)** *C*. *cigarro* CBS 237.49; **(C)** *C*. *cigarro* JKI-GP-23-030; **(D)** *C*. *cigarro* JKI-GP-23-019; **(E)** *C*. *cigarro* JKI-GP-23-031; **(F)** *C*. *cigarro* JKI-GP-23-020; **(G)** *C*. *gloeosporioides* s. str. DSM62136; **(H)** *C*. *gloeosporioides* s. str. DSM 62146; and **(I)** *C*. *gloeosporioides* s. str. CBS 119204. Bars: 20 µm.

Colony colors of *C. cigarro* isolates were variable, ranging from pale gray to pale brown, partly with yellow to orange clusters of conidia. Correspondingly, the reverse sides of the cultures were pale gray to pale brown. The average growth rate of the individual isolates ranged from 1.3 to 2.4 cm in 1 week (**Table 3**). Conidia were cylindrical (**Figure 3**) and variable in length (mean, 12.7–16.3 µm) and width (mean, 5.4–5.8 µm) (**Table 3**). Margin and aerial mycelium of the *C. gloeosporioides* isolates from other hosts were similar to those of the *C. cigarro* isolates. Colony colors were considerably variable as well, with conidiomata ranging from dark olive green (JKI-EP-62136) to orange (JKI-EP-62146, CBS 119204) depending on the conidia production.

**Table 3:** Mycelial growth and conidial dimensions of *Colletotrichum cigarro* and *C. gloeosporioides* on PDA after 7 days at 20°C in the dark.

Species	Strain	Mycelia growth (cm)	Conidia (*n* = 30)	
			Length (µm)	Width (µm)
*C. cigarro*	JKI-GF-Z1952	1.3 ± 0.0	(12.7−)12.9−14.4−15.9(−16.4)	(5.0−)5.1−5.8−6.4(−6.9)
*C. cigarro*	JKI-GP-23-030	2.4 ± 0.2	(11.6−)12.3−14.4−16.0(−16.5)	(4.8−)4.9−5.4−6.0(−6.6)
*C. cigarro*	JKI-GP-23-019	1.9 ± 0.2	(12.1−)12.6−14.1−15.8(−17.0)	(4.6−)5.2−5.8−6.4(−6.8)
*C. cigarro*	JKI-GP-23-031	1.8 ± 0.2	(10.0−)10.9−13.2−14.7(−15.3)	(4.9−)5.1−5.5−6.0(−6.1)
*C. cigarro*	JKI-GP-23-020	1.4 ± 0.1	(10.3−)11.0−12.7−13.9(−14.4)	(5.0−)5.1−5.7−6.4(−6.9)
*C. cigarro*	CBS 237.49	1.6 ± 0.0	(14.0−)14.5−16.3−17.8(−19.3)	(4.8−)5.2−5.7−6.2(−6.2)
*C. gloeosporioides* s. str.	DSM 62136	2.4 ± 0.0	(11.8−)13.2−14.6−16.0(−18.5)	(4.6−)5.4−6.1−6.7(−7.0)
*C. gloeosporioides* s. str.	DSM 62146	2.5 ± 0.0	(12.6−)12.6−13.7−14.8(−15.4)	(4.9−)5.3−5.9−6.5(−6.7)
*C. gloeosporioides* s. str.	CBS 119204	2.8 ± 0.1	(13.0−)13.5−15.0−16.1(−17.1)	(5.3−)5.4−6.2−6.7(−7.1)

The five values of conidia length and width indicate the minimum value, lower limit, arithmetic mean, upper limit, and maximum value, respectively; lower and upper limits indicate the range of 90% of all values.

### 3.4 Pathogenicity test

**Figure 4:**
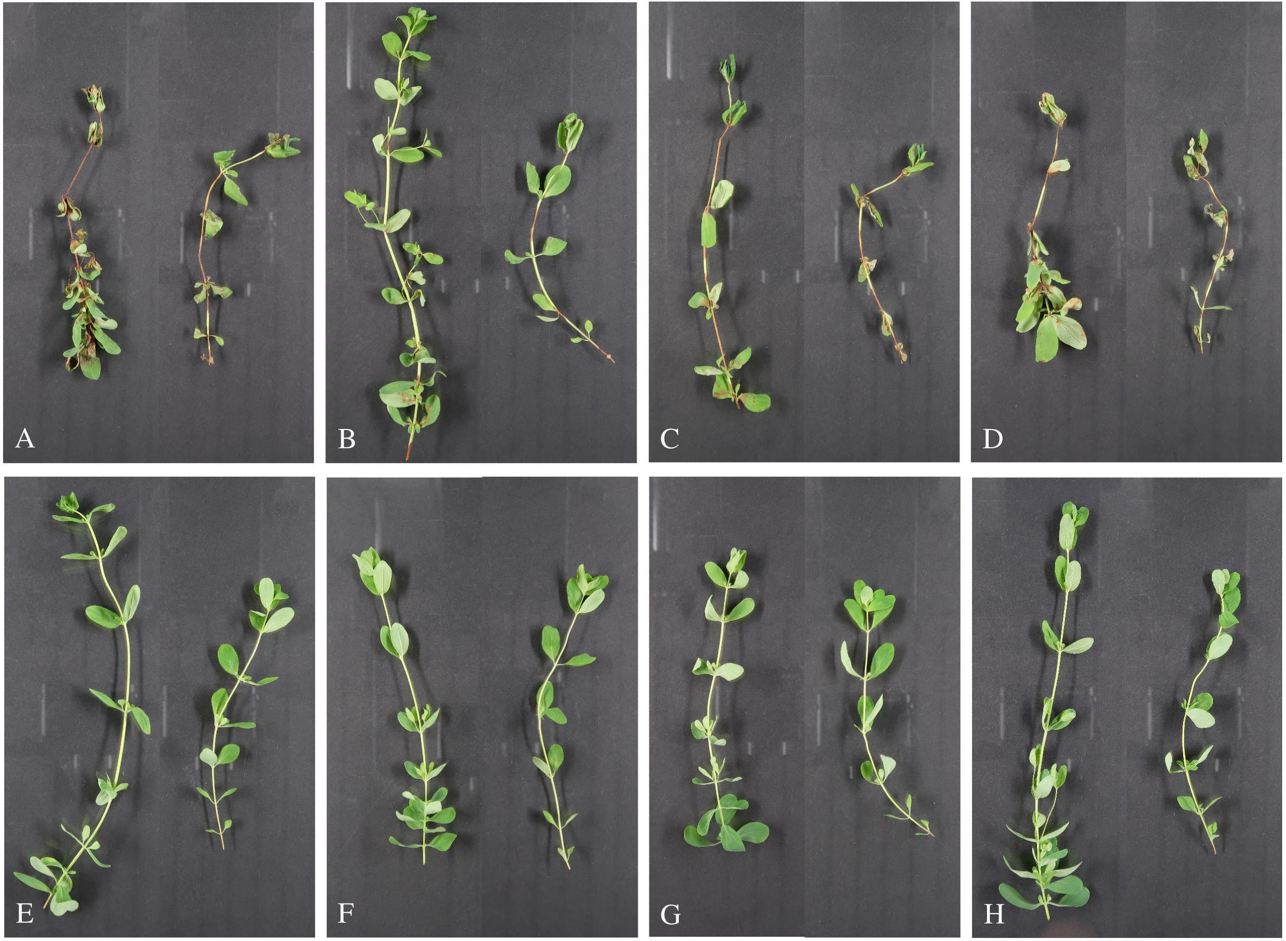
Pathogenicity test of *Colletotrichum cigarro* and *C*. *gloeosporioides* with *H*. *perforatum*. Representative head cuttings (left) and seedlings (right) harvested 1 week after inoculation with spore suspensions (1 × 10^6^ spores mL^−1^) of **(A)** *C*. *cigarro* JKI-GF-Z1952; **(B)** *C*. *cigarro* CBS 237.49; **(C)** *C*. *cigarro* JKI-GP-23-019; **(D)** *C*. *cigarro* JKI-GP-23-020; **(E)** *C*. *gloeosporioides* s. str. DSM 62136; **(F)** *C*. *gloeosporioides* s. str. DSM 62146; **(G)** *C*. *gloeosporioides* s. str. CBS 119204; and **(H)** non-inoculated control.

The first symptoms were observed on leaves and stems 4 days after inoculation with all *C. cigarro* strains from *H. perforatum*. The number and size of necrosis increased with time. Only minor differences were found in susceptibility between head cuttings and seedlings. First symptoms appeared 1–2 days earlier in seedlings than in head cuttings. Seven days after inoculation, differences between seedlings and head cuttings and between *C. cigarro* strains had diminished (**Figure 4**), and 14 days after inoculation, almost all plants inoculated with *C. cigarro* strains were completely wilted. From all symptomatic tissues collected randomly 7 days after inoculation, *C. cigarro* was re-isolated. In contrast to the plants inoculated with *C. cigarro*, no symptoms were visible on any of the young plants inoculated with *C. gloeosporioides* s. str. (**Figure 4**). However, this species was re-isolated from all randomly collected symptomless tissues 7 days after inoculation. No symptoms developed on the non-inoculated control plants, and no fungi were re-isolated from them. The repetition showed the same results.

## 4 Discussion

The major constrain in the cultivation of *H. perforatum* is St John’s wort wilt, which can cause severe to total crop losses (Gärber, 1999; Wahl et al., 2018). Despite the significant importance of *H. perforatum*, the causal organism of this disease had until now only been identified using morphological characters and was referred to as *C.* cf. *gloeosporioides* (Gärber, 1999) or *C. gloeosporioides* (Debrunner et al., 2000; Filoda, 2004; Michel et al., 2014). In this study, *Colletotrichum* spp. were isolated from symptomatic *H. perforatum* plants and seeds and from different locations and classified based on a comprehensive phylogenetic analysis. Initial BLASTn searches with ITS sequences of these strains on NCBI GenBank resulted in matches with strains of the *C. gloeosporioides* complex. This confirmed that the pathogen belongs to this species complex. Based on multi-locus phylogenetic analyses of ITS, GAPDH, ACT, and GS sequences, all *Colletotrichum* strains isolated from *H. perforatum* were clearly assigned to *C. cigarro*. This means that the St John’s wilt pathogen is not *C. gloeosporioides* as assumed in previous studies but belongs to the same species as the ex-type strain of *G. cingulata* var. *migrans*, CBS 237.49, which had previously been reassigned as *C. cigarro* (Weir et al., 2012; Cabral et al., 2020). The conidial size and growth rate were in agreement with those reported for *C. cigarro* by Weir et al. (2012) and for *G. cingulata* var. *migrans* by Wollenweber and Hochapfel (1949). However, they are also in agreement with many other species of the *C. gloeosporioides* and other species complexes (Weir et al., 2012; Damm et al., 2019). By comparing the individual isolates included in this study, a morphological variability was observed both within *C. cigarro* isolates from *H. perforatum* and within the *C. gloeosporioides* s. str. isolates included in this study. On the basis of conidial shape and size and colony characteristics, it would not have been possible to assign the isolates to either of these species. Rose and Damm (2024) showed that morphological data were not sufficient to identify *Colletotrichum* species from strawberries belonging to the *C. acutatum* and *C. dematium* complexes. The data from this study confirm that identification of *Colletotrichum* species based on morphological characteristics alone is not possible. For an unambiguous species assignment, DNA sequence data are necessary. Despite the variable morphology, the sequences of the four loci of all eight *C. cigarro* isolates from *H. perforatum* examined morphologically were identical, regardless of country, location, and year of collection, including those of the nearly 90-year-old isolate CBS 237.49. The pathogen apparently had not changed over time and can probably be considered as a clonal lineage. The ApMat sequences of selected *C. cigarro* strains generated in this study support this hypothesis as they are identical (data not shown). Clonal spread has also been observed in *C. nymphaeae* from strawberries in Germany and other countries that are in connection with global trade (Rose and Damm, 2024). However, until now, there are only data available of strains from Germany and Switzerland, two countries within Central Europe. Sequence data of strains from other regions would enhance our knowledge about the disease.

In this study, no further *Colletotrichum* species were detected on *H. perforatum*. In the USDA Fungal Databases (https://fungi.ars.usda.gov; last visited 23 October 2024), there was also no further species listed that had been identified based on sequence data. The identification of the St John’s wort pathogen from Hungary as *C. gloeosporioides* was based on morphology (Schwarczinger and Vajna, 1998) and is therefore not reliable. Moreover, an unidentified *Colletotrichum* species had been reported from stem anthracnose of *H. graveolens*, *H. michellianum*, *H. perforatum*, and *H. punctatum* in the USA (Grand, 1985). Both reports could refer to *C. cigarro*, which cannot be confirmed due to the lack of sequence data. In contrast, DNA sequences of further *Colletotrichum* isolates from *Hypericum* were detected in the NCBI database (Nirenberg et al., 2002; Ellsworth et al., 2013; Staňková et al., 2014; Samaga and Rai, 2016; Ding et al., 2023). These sequences were included in the analyses of the present study. Only the *C.* “*gloeosporioides*” isolate BBA 70048 (from *Hypericum* sp., without location) clustered in the ITS phylogeny with the *C. cigarro* isolates from *H. perforatum* sequenced in this study, however, also with other species that have identical ITS sequences. The ITS sequences of further Colletotrichum isolates from *Hypericum* found in GenBank that were previously identified as *C. kahawae, C. gloeosporioides, C. acutatum* and *C. graminicola* were isolated from symptom less *Hypericum* spp. plants in different countries (Ellsworth et al., 2013; Staňková et al., 2014; Samaga and Rai, 2016; Ding et al., 2023); they either clustered in the ITS phylogeny with other species of the *C. gloeosporioides* complex or belong to species of other species complexes. As these sequences were from the ITS region, it was not possible to identify the isolates to species level with certainty; sequence data of loci with a higher resolution are necessary to identify them. Since these fungi were isolated as endophytes, it is not very likely that they cause St John’s wilt and even less likely that they pose a major threat to *Hypericum* cultivation.

To confirm that *C. cigarro* causes St John’s wilt of *H. perforatum*, pathogenicity tests were carried out with selected strains of *C. cigarro* from *H. perforatum* and for comparison with isolates of *C. gloeosporioides* s. str. from other hosts on seedlings and head cuttings of St John’s wort. All strains of *C. cigarro* caused severe anthracnose with subsequent wilt symptoms on *H. perforatum* plantlets. It is particularly noteworthy that the symptoms of the strains isolated in this study corresponded to those of the strain CBS 237.49, isolated in 1937 (Wollenweber and Hochapfel, 1949). Interestingly, plantlets inoculated with *C. gloeosporioides* s. str. showed no symptoms but were latently infected. The same was observed after inoculation of *H. perforatum* with strains of *C. nymphaeae* and *C. destructivum* (data not shown) belonging to the *C. acutatum* and *C. destuctivum* complexes, respectively, and are common on other hosts in Europe (Damm et al., 2012, 2014; Rose and Damm, 2024). This indicates that other species of the genus can infect *H. perforatum* but do not cause symptoms and could explain the isolations of, e.g., representatives of the *C. acutatum* and *C. graminicola* species complex from *H. perforatum* in the Czech Republic (Staňková et al., 2014). If host plants of these species complexes are nearby, latent *H. perforatum* infection may be possible.

Based on this study, St John’s wilt of *H. perforatum* in Europe is caused only by *C. cigarro*. However, different *Colletotrichum* species might be latently present in *H. perforatum* in Europe and other regions of the world, and further *Hypericum* species might also be affected by other *Colletotrichum* species. To address these points and answer the question of whether *C. cigarro* isolates from other host plants could also cause symptoms on *H. perforatum*, further studies are needed.

## Data Availability

The datasets presented in this study can be found in online repositories. The names of the repository/repositories and accession number(s) can be found in the article/**Supplementary Material**.
