# Supplementary figures and images for: A new name for an old problem—Colletotrichum cigarro is the cause of St John’s wilt of Hypericum perforatum
Source: Front Fungal Biol. 2025 Jan 23;5:1534080. doi: 10.3389/ffunb.2024.1534080 (PMC11799269; doi:10.3389/ffunb.2024.1534080)

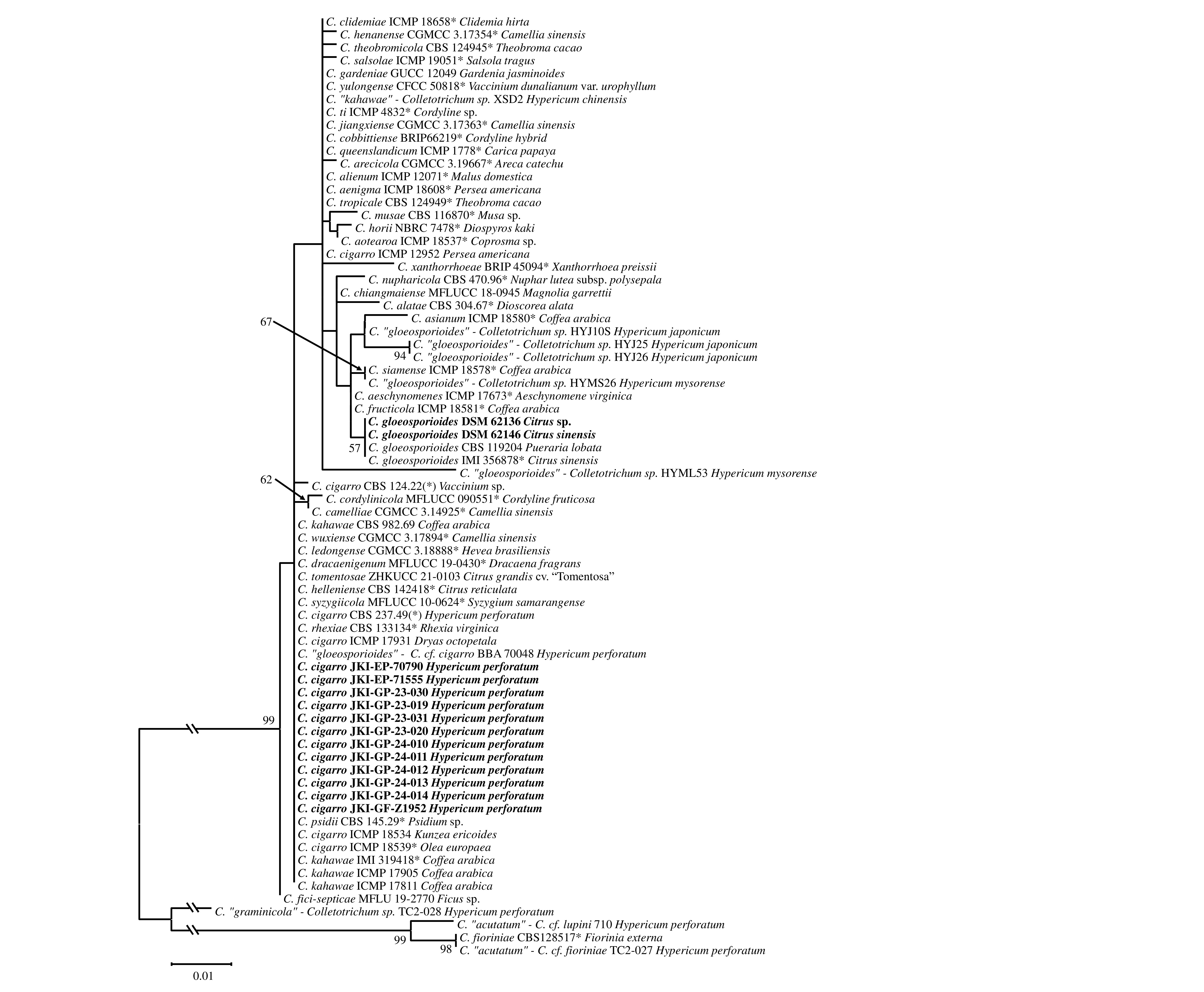

Supplement: Supplementary file 1 [file Image1.jpeg]

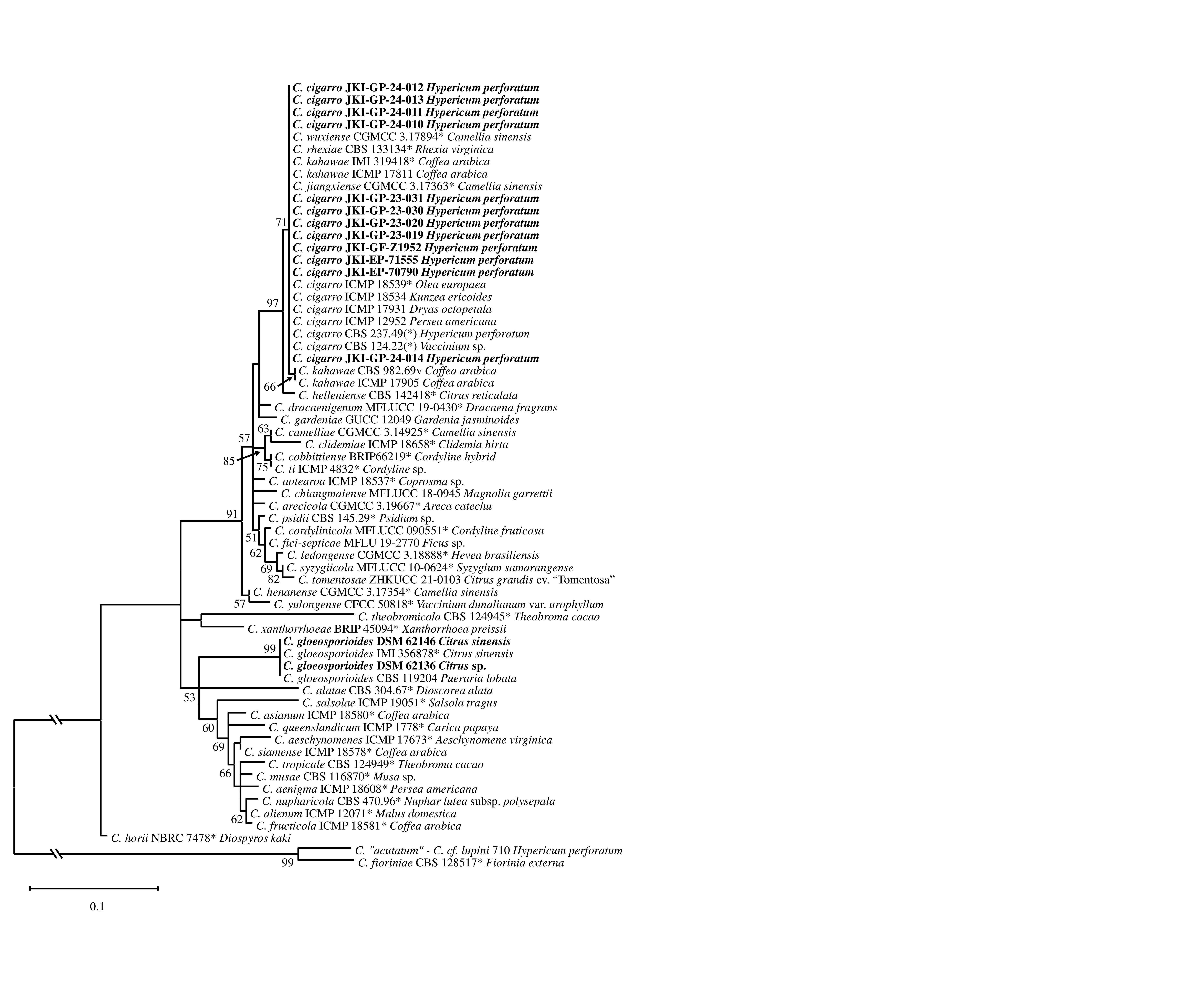

Supplement: Supplementary file 2 [file Image2.jpeg]

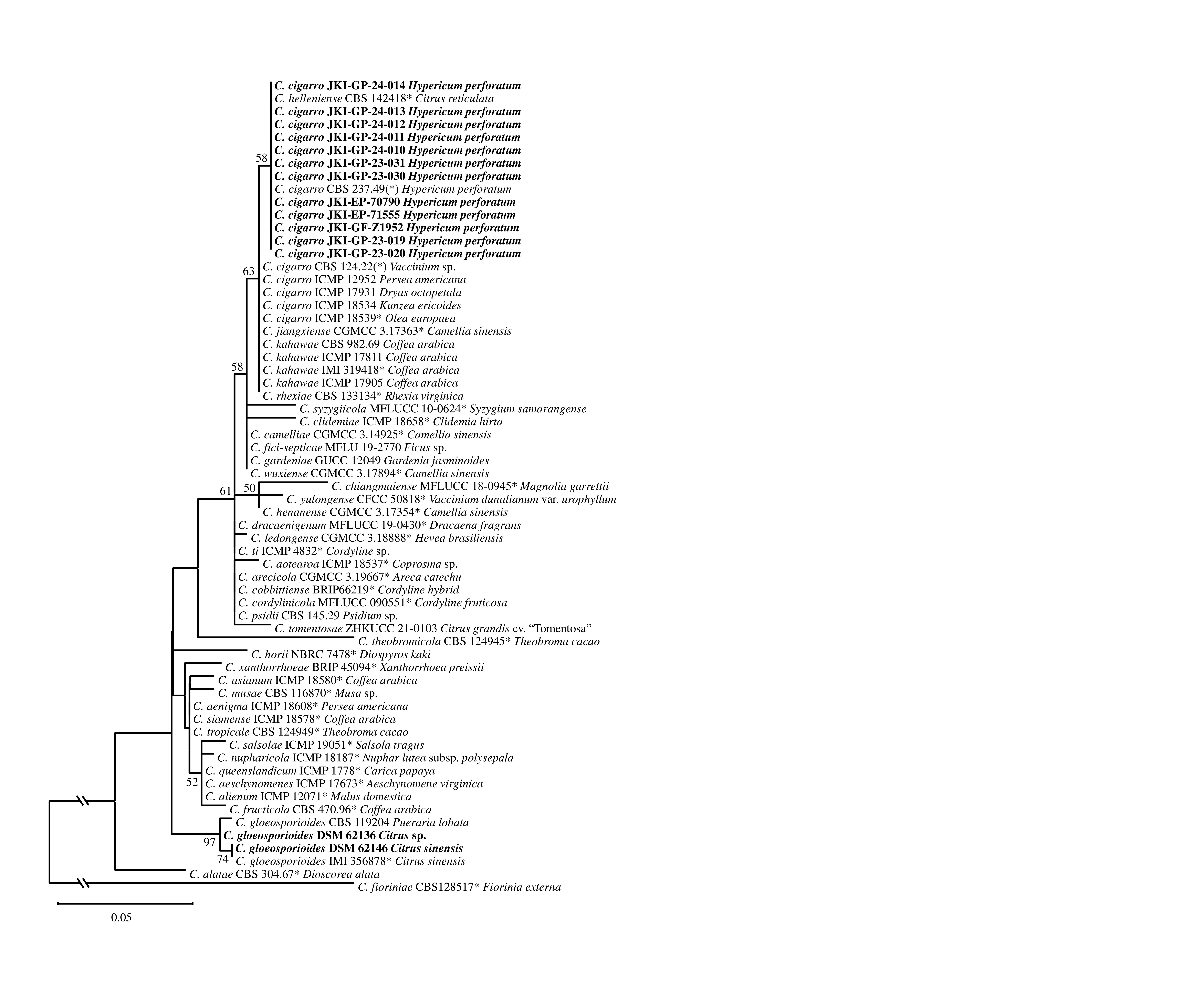

Supplement: Supplementary file 3 [file Image3.jpeg]

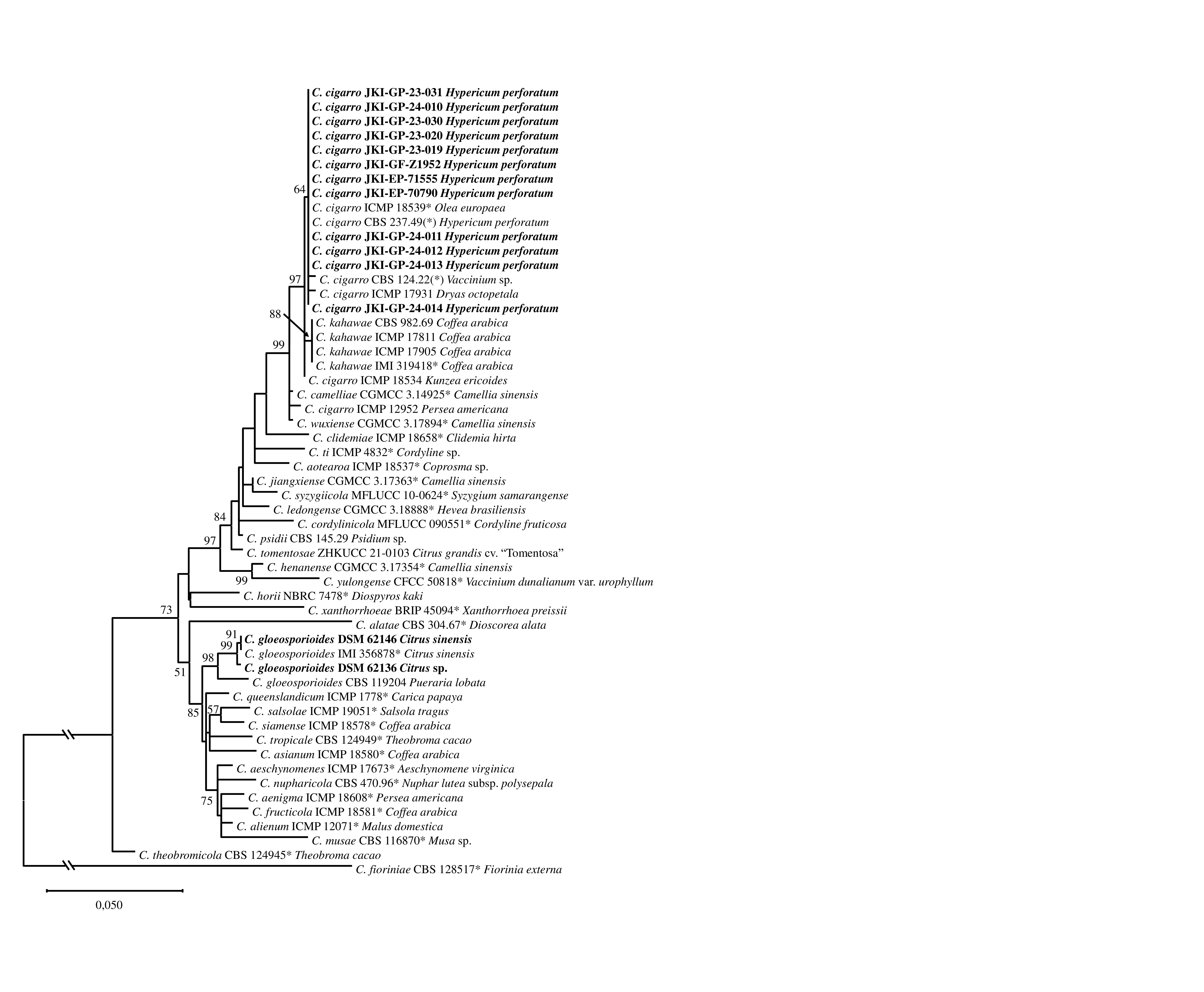

Supplement: Supplementary file 4 [file Image4.jpeg]
