# Supplementary material for: A new name for an old problem—Colletotrichum cigarro is the cause of St John’s wilt of Hypericum perforatum
Source: Front Fungal Biol. 2025 Jan 23;5:1534080. doi: 10.3389/ffunb.2024.1534080 (PMC11799269; doi:10.3389/ffunb.2024.1534080)
Supplement: Supplementary file 6 [file Table2.docx]

Supplementary Material

# Supplementary Figures and Tables

Supplementary Figure 1 Maximum likelihood phylogenetic tree based on ITS sequences of *Colletotrichum* species in the *C. gloeosporioides* species complex. Numbers above branches show bootstrap values ≥ 50. Strains obtained in this study are shown in bold. Ex-type strains are emphasized with an asterisk. Scale bar: number of substitutions per site.

Supplementary Figure 2 Maximum likelihood phylogenetic tree based on GAPDH sequences of *Colletotrichum* species in the *C. gloeosporioides* species complex. Numbers above branches show bootstrap values ≥ 50. Strains obtained in this study are shown in bold. Ex-type strains are emphasized with an asterisk. Scale bar: number of substitutions per site.

Supplementary Figure 3 Maximum likelihood phylogenetic tree based on ACT sequences of *Colletotrichum* species of the *C. gloeosporioides* species complex. Numbers above branches show bootstrap values ≥ 50. Strains obtained in this study are shown in bold. Ex-type strains are emphasized with an asterisk. Scale bar: number of substitutions per site.

Supplementary Figure 4 Maximum likelihood phylogenetic tree based on GS sequences of *Colletotrichum* species of the *C. gloeosporioides* species complex. Numbers above branches show bootstrap values ≥ 50. Strains obtained in this study are shown in bold. Ex-type strains are emphasized with an asterisk. Scale bar: number of substitutions per site.

**Supplementary Table 1** Accession numbers of isolates included in this study. Ex-type strains are emphasized with an asterisk.
